# Supplementary material for: Distinct Perception Mechanisms of BACH1 Quaternary Structure Degrons by Two F-box Proteins under Oxidative Stress
Source: bioRxiv. 2024 Jun 3:2024.06.03.594717. Preprint. [Version 1] doi: 10.1101/2024.06.03.594717 (PMC11185555; doi:10.1101/2024.06.03.594717)
Supplement: Supplement 1 [file NIHPP2024.06.03.594717v1-supplement-1.pdf]

bioRxiv preprint doi: https://doi.org/10.1101/2024.06.03.594717; this version posted June 3, 2024. The copyright holder for this preprint (which was not certified by peer review) is the author/funder, who has granted bioRxiv a license to display the preprint in perpetuity. It is made available under aCC-BY 4.0 International license.

Supplementary Materials

Cao S. *et al.* 2023

Supplementary Figure Legends

Supplementary Figure 1. The schematic workflow of single particle reconstruction of the $SCF^{FBXO22\text{-}BACH1}$complex.

Supplementary Figure 2. Sequence and structural analysis of FBXO22.

Supplementary Figure 3. Structural and biochemical analyses of FBXO22-BACH1 interaction and sequence analysis of NCOR1/2.

Supplementary Figure 4. BLI analysis of FBXL17-BACH1-BTB interactions and the schematic workflow of single particle reconstruction of the $SCF^{FBXL17\text{-}BACH1}$complex.

Supplementary Figure 5. Cryo-EM single particle analysis workflow of wild type $SCF^{FBXL17\text{-}BACH1}$.

Supplementary Table 1. Cryo-EM data collection, refinement, and validation statistics

bioRxiv preprint doi: https://doi.org/10.1101/2024.06.03.594717; this version posted June 3, 2024. The copyright holder for this preprint (which was not certified by peer review) is the author/funder, who has granted bioRxiv a license to display the preprint in perpetuity. It is made available under aCC-BY 4.0 International license.

Supplementary Figure Legends

Figure S1. The schematic workflow of single particle reconstruction of the SCF$^{FBXO22-BACH1}$complex. A. A representative cryo-EM micrograph. **B.** Typical 2D averages of the cryo-EM dataset. Scale bar 10 nm. **C.** The flowchart of single particle analysis of the SCF$^{FBXO22-BACH1}$complex. **D.** The angular distribution of particles used in the final reconstruction. **E,** Fourier shell correlation (FSC) curves for SCF$^{FBXO22-BACH1}$. At the Gold-standard threshold of 0.143, the resolution is 3.9 Å. **F.** Representative density in local refined EM map.

Figure S2. Sequence and structural analysis of FBXO22. A. Sequence alignment of five vertebrate FBXO22 orthologues with second structure annotations. The sequences of the three FIST domains are underlined in different colors (slate, salmon, and purple). Unique secondary structure elements in each repeat are highlighted in red. **B.** A comparison of the three FIST domains of FBXO22 and homologous structure of YabJ from the YjgF superfamily (PDB:1QD9).

Figure S3. Structural and biochemical analyses of FBXO22-BACH1 interaction and sequence analysis of NCOR1/2. A. Steric hindrance prevents the formation of a BACH1-BTB-FBXO22 complex with a 2:2 ratio. The asymmetric complex formed between a BACH1 dimer (protomer A: light blue; subunit B: orange) and FBXO22 (purple) is shown as cartoon diagram. A second copy of FBXO22 shown in slate surface representation is modeled onto the BACH1-BTB dimer and is in clash with the other FBXO22 macromolecule. **B.** *In vitro* ubiquitination of BACH1 by SCF^{FBXO22}. **C.** Superposition of the crystal structures of BACH1-BTB (PDB:2IHC) and BACH2-BTB (PDB:3OHU). BACH2 C-terminal region is disordered and highlighted in red. **D.** Sequence alignment of five NCOR1 vertebrate orthologs and human NCOR2. Highly conserved short linear motifs (SLiMs) are underlined with gray bars. The BCL6 BTB-interacting motif found in PDB:1R2B is underlined with a purple bar.

Figure S4. BLI analysis of FBXL17-BACH1-BTB interactions and the schematic workflow of single particle reconstruction of the SCF$^{FBXL17-BACH1}$complex. A. BLI measurements of the binding between FBXL17 and NOR3-S1PC-treated BACH1-BTB with a 10-minutes association step. K_d, dissociation constant. **B. & C.** BLI measurements of the interaction between FBXL17 and BACH1-BTB (wild type and C34A mutant) untreated or treated with NOR3-S1PC. In the absence of compound treatment, C34A enhanced FBXL17 binding. This effect is exaggerated upon compound treatment. The C34 residue, therefore, is not required for S-nitrosylation. **D.** A representative cryo-EM micrograph for the sample containing a mixture of SCF^{FBXL17} and BACH1-BTB treated with NOR3-S1PC. **E.** Typical 2D averages. **F.** The flowchart of single particle analysis of the sample containing a mixture of SCF^{FBXL17} and BACH1-BTB treated with NOR3-S1PC.

Figure S5. Cryo-EM single particle analysis workflow of wild type SCF$^{FBXL17-BACH1}$. A. A representative cryo-EM micrograph. **B.** Typical 2D averages revealing the most populated monomeric complex. **C.** The flowchart of single particle analysis of the sample containing a mixture of SCF^{FBXL17} and BACH1-BTB. **D.** The particle angular distribution and FSC curves of dSCF$^{FBXL17-BACH1-I}$ with local refinement. **E**. The particle angular distribution and FSC curves of dSCF$^{FBXL17-BACH1-II}$. **F.** The angular distribution and FSC curves of the monomeric SCF$^{FBXL17-BACH1}$ complex.

bioRxiv preprint doi: https://doi.org/10.1101/2024.06.03.594717; this version posted June 3, 2024. The copyright holder for this preprint (which was not certified by peer review) is the author/funder, who has granted bioRxiv a license to display the preprint in perpetuity. It is made available under aCC-BY 4.0 International license.

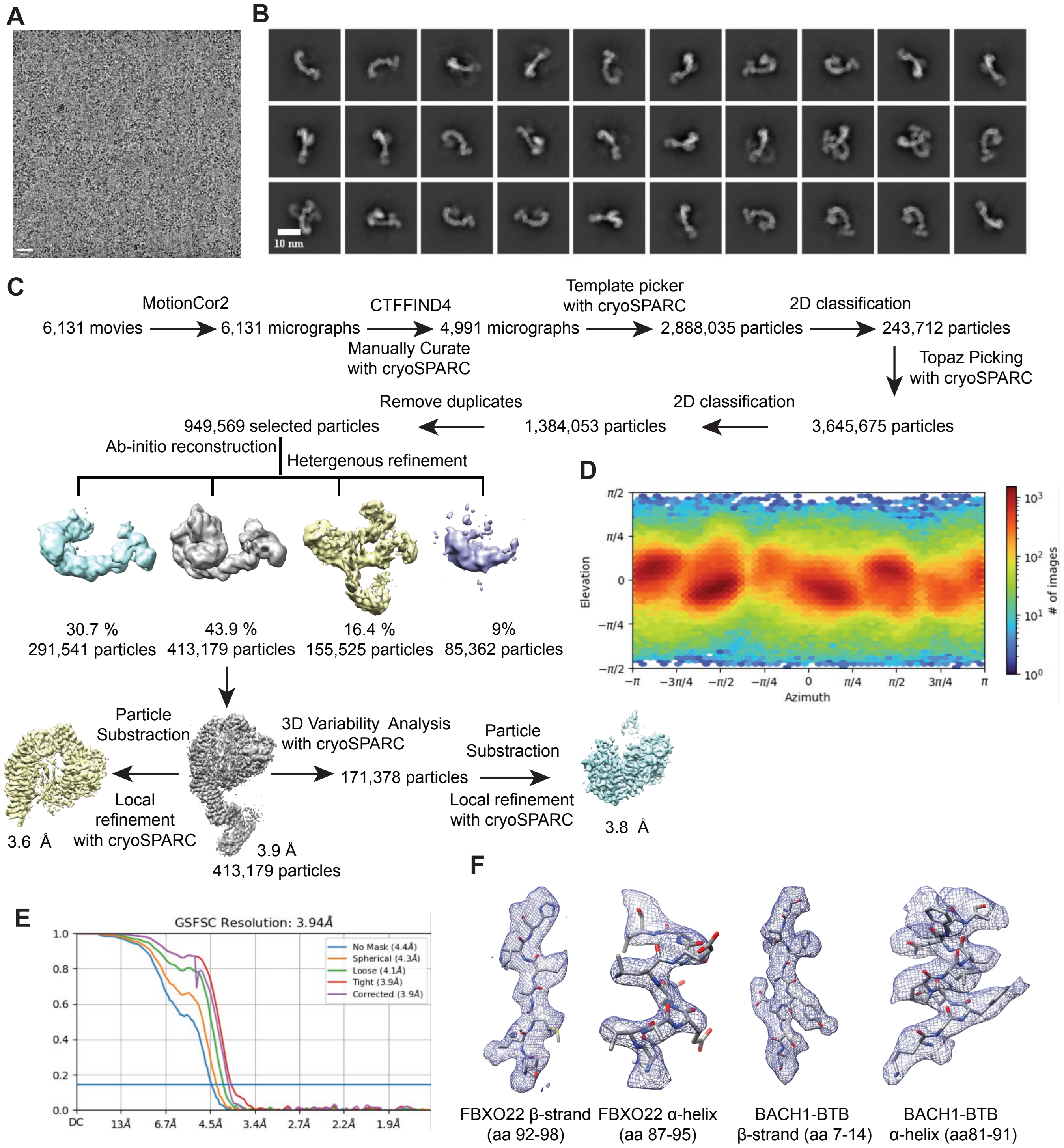

Figure S1

bioRxiv preprint doi: https://doi.org/10.1101/2024.06.03.594717; this version posted June 3, 2024. The copyright holder for this preprint (which was not certified by peer review) is the author/funder, who has granted bioRxiv a license to display the preprint in perpetuity. It is made available under aCC-BY 4.0 International license.

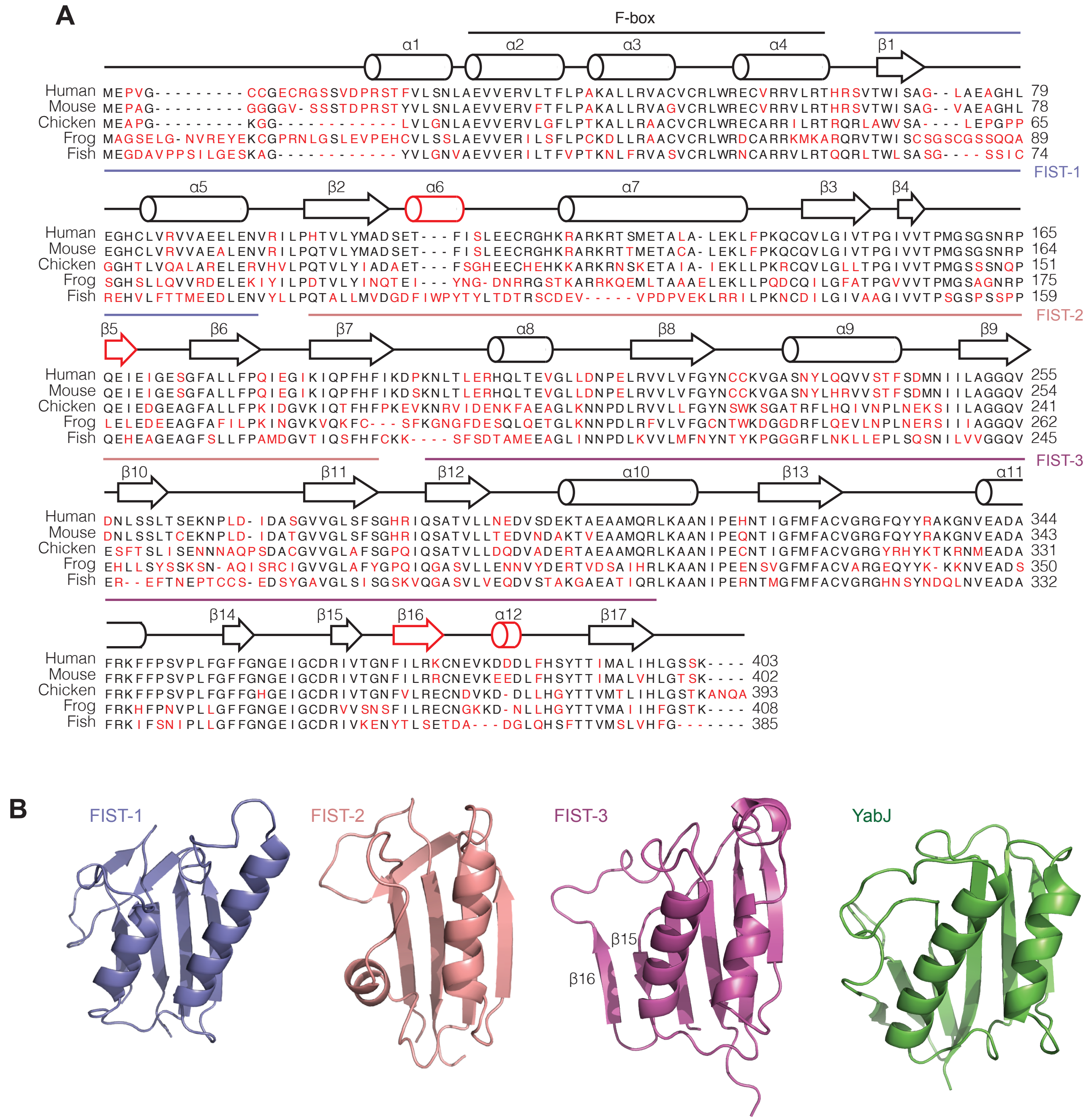

Figure S2

bioRxiv preprint doi: https://doi.org/10.1101/2024.06.03.594717; this version posted June 3, 2024. The copyright holder for this preprint (which was not certified by peer review) is the author/funder, who has granted bioRxiv a license to display the preprint in perpetuity. It is made available under aCC-BY 4.0 International license.

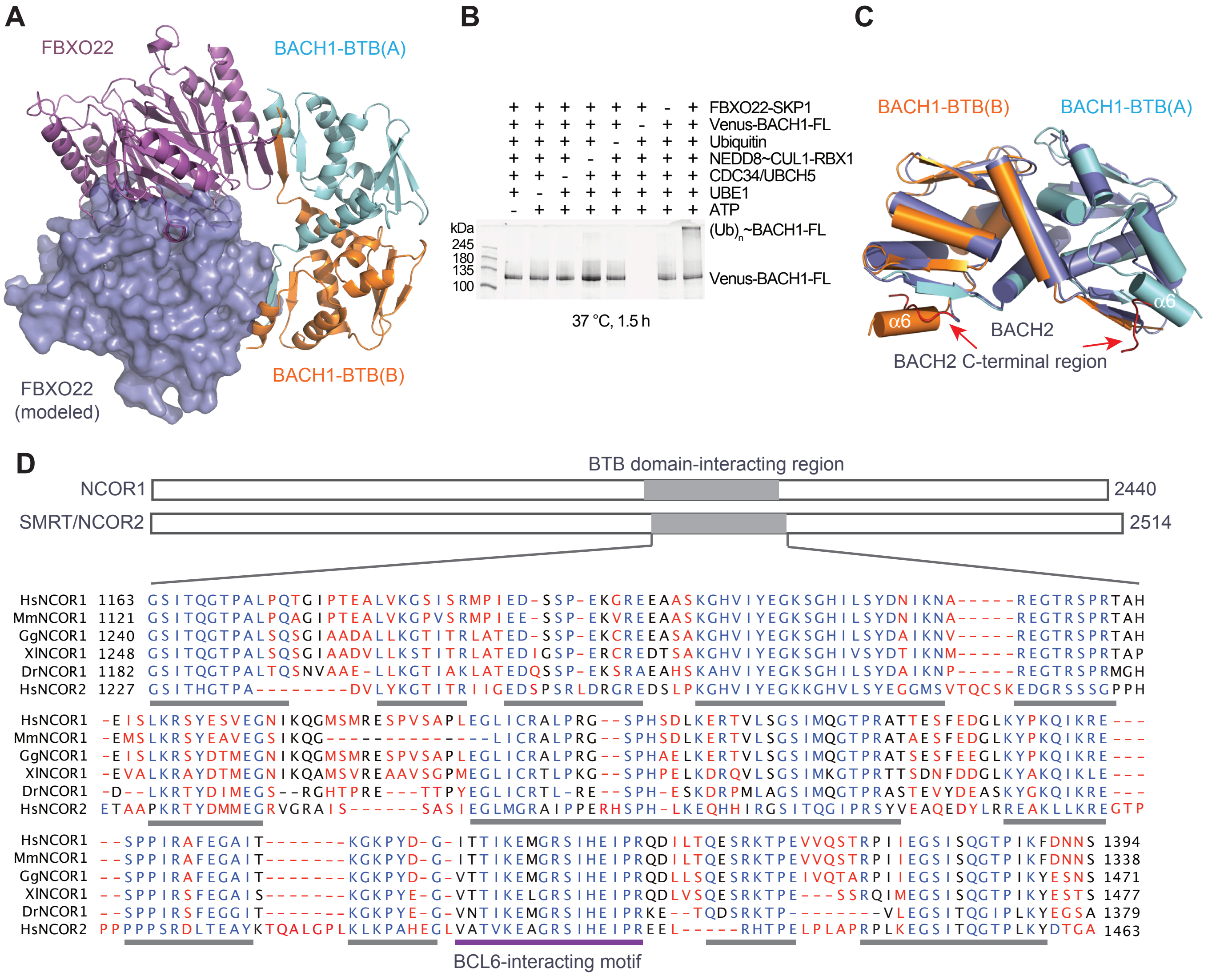

Figure S3

bioRxiv preprint doi: https://doi.org/10.1101/2024.06.03.594717; this version posted June 3, 2024. The copyright holder for this preprint (which was not certified by peer review) is the author/funder, who has granted bioRxiv a license to display the preprint in perpetuity. It is made available under aCC-BY 4.0 International license.

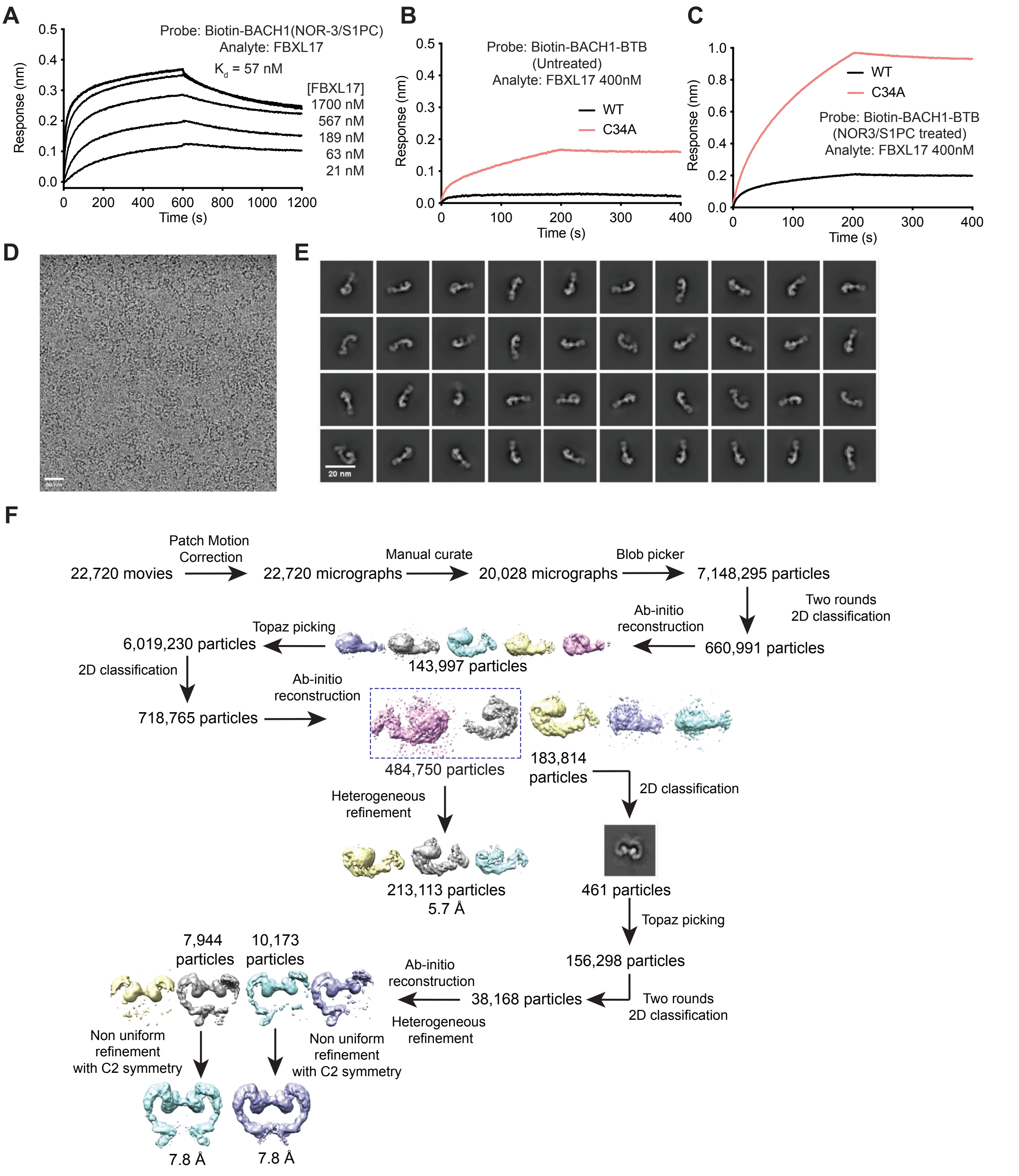

Figure S4

bioRxiv preprint doi: https://doi.org/10.1101/2024.06.03.594717; this version posted June 3, 2024. The copyright holder for this preprint (which was not certified by peer review) is the author/funder, who has granted bioRxiv a license to display the preprint in perpetuity. It is made available under aCC-BY 4.0 International license.

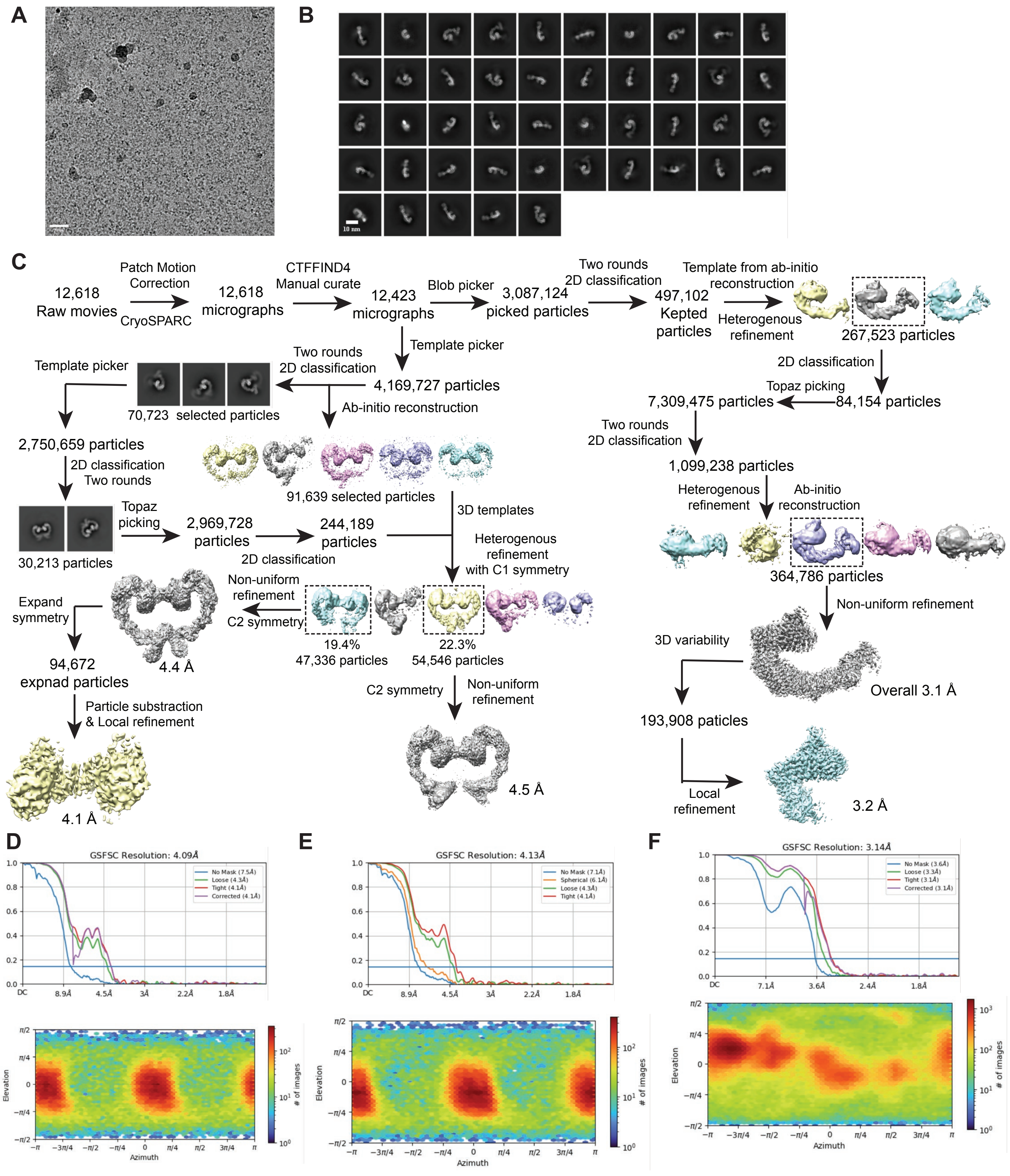

Figure S5

bioRxiv preprint doi: https://doi.org/10.1101/2024.06.03.594717; this version posted June 3, 2024. The copyright holder for this preprint (which was not certified by peer review) is the author/funder, who has granted bioRxiv a license to display the preprint in perpetuity. It is made available under aCC-BY 4.0 International license.

Table S1. Cryo-EM data collection, refinement, and validation statistics

	**SCF^{FBXO22}-BACH1-BTB**	**SCF^{FBXL17}-BACH1-BTB Monomer**	**SCF^{FBXL17}-BACH1-BTB Dimer**
**Data collection and processing**			
Magnification	105,000	165,000	165,000
Voltage (kV)	300	300	200
Electron exposure (e^{-}/Å^2)	59	60	60
Defocus range (μm)	-1.8~-3.5	-0.8~-2.5	-0.8~-2.5
Pixel size (Å)	0.84	0.743	0.743
Symmetry imposed	C1	C1	C2
Initial particle images (no.)	2,888,035	3,087,124	2,969,728
Final particle images (no.)	413,523	364,786	71,155
Map resolution (Å)	3.9	3.1	4.1
FSC threshold	0.143	0.143	0.143
Map resolution range (Å)	3.9~6.5	3.1~6.5	4.1~7.0
**Refinement**			
Initial model	AlphaFold 2	6WCQ/1LDJ	SCF-FBXL17-BACH1-BTB
Map sharpening *B* factor (Å^2)	-243.4	-99.6	-145.5
Model composition			
Non-hydrogen atoms	9,822	8,798	23,028
Protein residues	1,232	1,101	2861
Ligands	-	-	-
Zn^{2+}	-	-	6
B-factors (Å^2)			
Protein	85.24	28.12	17.28
Ligand	-	-	15.57
R.m.s. deviations			
Bond lengths (Å)	0.005	0.004	0.0044
Bond angles (°)	1.078	0.809	0.823
Validation			
MolProbity score	2.25	2.28	2.19
Clashscore	13.68	10.25	12.59
Poor rotamers (%)	0	0	0.1
Ramachandran Plot			
Favored (%)	87.73	86.2	90.21
Allowed (%)	11.53	13.16	9.61
Disallowed (%)	0.74	0.64	0.18
